# Potential for Novel Biomarkers in Diabetes-Associated Chronic Kidney Disease: Epigenome, Metabolome, and Gut Microbiome

**DOI:** 10.3390/biomedicines8090341

**Published:** 2020-09-10

**Authors:** Ashani Lecamwasam, Elif I. Ekinci, Richard Saffery, Karen M. Dwyer

**Affiliations:** 1 Epigenetics Group, Murdoch Children’s Research Institute, Parkville, VIC 3052, Australia; richard.saffery@mcri.edu.au; 2 Department of Endocrinology, Austin Health, Ivanhoe, VIC 3079, Australia; elif.ekinci@unimelb.edu.au; 3 School of Medicine, Faculty of Health, Deakin University, Geelong Waurn Ponds, VIC 3220, Australia; karen.dwyer@deakin.edu.au; 4 Department of Medicine, University of Melbourne, Parkville, VIC 3010, Australia; 5 Department of Paediatrics, University of Melbourne, Parkville, VIC 3010, Australia

**Keywords:** diabetes, chronic kidney disease, biomarker, epigenetics, metabolomics, gut microbiome

## Abstract

Diabetes-associated chronic kidney disease is a pandemic issue. Despite the global increase in the number of individuals with this chronic condition together with increasing morbidity and mortality, there are currently only limited therapeutic options to slow disease progression. One of the reasons for this is that the current-day “gold standard” biomarkers lack adequate sensitivity and specificity to detect early diabetic chronic kidney disease (CKD). This review focuses on the rapidly evolving areas of epigenetics, metabolomics, and the gut microbiome as potential sources of novel biomarkers in diabetes-associated CKD and discusses their relevance to clinical practice. However, it also highlights the problems associated with many studies within these three areas—namely, the lack of adequately powered longitudinal studies, and the lack of reproducibility of results which impede biomarker development and clinical validation in this complex and susceptible population.

## 1. The Need for Improved Biomarkers in Diabetes-Associated Chronic Kidney Disease

What defines a biomarker? In 1998, the National Institutes of Health Biomarkers Definitions Working Group defined a biomarker as “a characteristic that is objectively measured and evaluated as an indicator of normal biological processes, pathogenic processes, or pharmacologic responses to a therapeutic intervention” [1]. The World Health Organization (WHO), in their report on the validity of biomarkers in environment risk assessment, state that a purist definition of biomarkers includes “almost any measurement reflecting an interaction between a biological system and a potential hazard, which may be chemical, physical, or biological. The measured response may be functional and physiological, biochemical at the cellular level, or a molecular interaction” [2]. Of course, good biomarkers need to be specific, sensitive, and detectable in specimens obtained through minimally invasive procedures to be clinically useful [3]. Some examples of biomarkers include the spectrum of clinical parameters, such as blood pressure measurements; biochemical measures, such as serum troponin for cardiac disease; and more complex laboratory investigations of blood and other tissues.

Despite the global increase in the number of individuals with diabetes and diabetes-associated chronic kidney disease (CKD) [4], there are currently only limited therapeutic options to slow the progression of this chronic condition. One of the reasons for this is the lack of sensitive markers to identify early diabetic CKD, thus impeding clinical intervention to prevent progression and associated morbidity and mortality. The current gold-standard biomarkers to evaluate kidney function include serum creatinine. Unfortunately, serum creatinine is influenced by a number of factors, including an individual’s muscle mass, state of hydration, diet, and concurrent medication use, resulting in a poor sensitivity and specificity. In fact, serum creatinine does not significantly increase until the glomerular filtration rate (GFR) is reduced to half of its normal level [5], thus leading to underestimations of the true GFR. The estimated glomerular filtration rate (eGFR), which is based on the serum creatinine concentration, also takes into account age and gender [6,7]; it was developed to address some of the above limitations, but has its own inherent problems. This is primarily because eGFR is subject to variation due to the analytical error of the creatinine measurement and biological variation [5]. Consequently, CKD, including diabetic CKD, is often undetected until substantial kidney injury has occurred.

The stage of CKD is dependent on two components: the eGFR and the urine Albumin-to-Creatinine Ratio (uACR). Microalbuminuria is defined as an uACR of 30–300 mg/g and macro albuminuria is defined as an uACR >300 mg/g [8]. The traditional paradigm of diabetes-associated CKD is that of a progressive increase in urinary albumin excretion combined with a drop in eGFR. However, several studies have shown that even with normal urinary albumin excretion, the eGFR may be severely reduced [9,10] and, conversely, individuals with significantly elevated urinary albumin excretion or proteinuria can have a normal eGFR. Importantly, both eGFR and uACR are independently associated with progressive renal dysfunction, cardiovascular disease, and death [11]. With our current tools, it is not possible to identify or predict which individuals will rapidly decline in kidney function compared to those who may have a slower decline.

**Figure 1:**
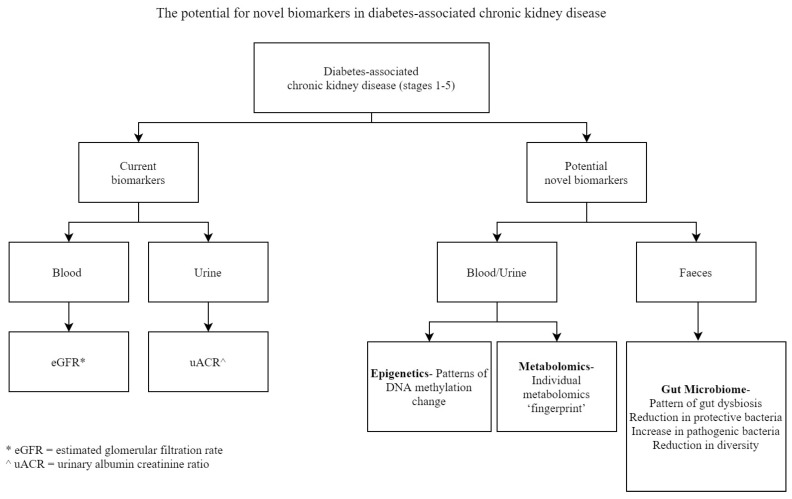
The potential for novel biomarkers in diabetes-associated chronic kidney disease.

One of the other significant elements is, of course, determining the relationship between a measurable biomarker and its relevant clinical endpoints. As pointed out by Kit et al., traditionally, proteins either in the plasma or serum have been used as biomarkers to identify individuals with an increased risk of developing disease, screening for early detection, or evaluating the response to treatment [3]. However, more recently RNA or DNA biomarkers together with small molecules such as metabolites have been identified as potential biomarkers in diabetic and non-diabetic CKD [12,13,14], Figure 1. This is encouraging, but there needs to be caution in confirming and replicating these results in larger, longitudinal cohorts before its validation in clinical practice. Diabetes-associated CKD is a multifactorial disease with risk factors inclusive of ethnicity, hyperglycemia, hypertension, obesity, and dyslipidemia. Glucose and blood pressure-lowering medications and even multifactorial lifestyle interventions have modest effects on reducing the decline in kidney function [15]. New strategies are therefore required if we are to collectively reduce the burden of this chronic, multisystemic, and multifactorial disease. Therefore, biomarker research with potential clinical translation should look at novel emerging areas such as epigenetics, metabolomics, and the gut microbiome. This review will focus on the potential for biomarkers identified through epigenetic, metabolomic, and gut microbiome studies in diabetes-associated CKD and discuss their relevance to clinical practice.

## 2. Epigenetic Biomarkers in Diabetes-Associated Chronic Kidney Disease

Epigenetics refers to the “structural adaptation of chromosomal regions so as to register, signal or perpetuate altered activity states” [16] and, consequently, is considered to be the interface between the genotype and phenotype. It helps to explain why some genetic changes do not always lead to an altered phenotype. As such, in addition to genomic and transcriptomic analyses, epigenomic biomarkers provide insight into the role of different pathways in disease [17]. The epigenetic regulation of gene expression impacts the availability of target gene mRNA available for translation, thus affecting protein synthesis. Evidence suggests that the loss of this epigenetic regulation and the differential DNA methylation detected in kidney fibrogenesis may be associated with CKD [18].

### 2.1. DNA Methylation as a Biomarker

DNA methylation is the transfer of a methyl group (-CH3) from S-adenosylmethionine to the fifth carbon of a cytosine nucleotide in the DNA sequence, catalyzed by DNA methyltransferases (DNMTs) [19]. The presence of such methyl groups leads to an inhibition of gene transcription and therefore an absence of gene expression. Once methylation is acquired, it is in most cases chemically and biologically stable over time. Given the stability of these methyl groups and the amplifiable and stable nature of DNA, it can be used with relative ease from research to diagnostic environments [3]. An important criterion of biomarkers is the ability to analyze a clinically relevant marker from biological specimens that are obtained via minimally invasive procedures. Urine can be collected rapidly and easily as a non-invasive biological specimen and contains cells reflective of the kidneys. Thus, the detection of methylation in urine would offer an enticing approach for measuring DNA methylation profiles directly related to diabetic chronic kidney disease. However, we have shown that the yield of genomic DNA may be limited for the purposes of identification of urine-based DNA methylation biomarkers in diabetes-associated CKD [20]. 

There have, however, been multiple studies looking at other sources of DNA, such as blood, and evaluating the methylation patterns associated with diabetic kidney disease [21,22,23]. It should be noted that the analysis of DNA methylation patterns is complicated by the fact that some changes are due to environmental influences, as well as effects of ageing at some promoter sites [3]. To be useful as biomarkers, diabetes-associated chronic kidney disease alterations have to be distinguishable from age-associated DNA methylation changes. A further challenge with DNA methylation as a biomarker is to identify single biomarkers with sufficient sensitivity and specificity. Experimental variation can be a problem if degraded samples, such as those that are formalin-fixed or paraffin-embedded, are used for analysis [3]. Furthermore, it is important that there is analysis of more than a single CpG site to obtain reliable results, especially if the gene is heterogeneously methylated with the co-existence of many different alleles [24]. 

### 2.2. Emerging Trends in Epigenetic Modification Analysis

Understanding how DNA methylation influences chromatin function and its role in both normal development and pathological states requires the ability to analyze DNA methylation patterns of the entire genome. The methodologies to enable this have considerably increased over the past decade, and several approaches have been developed to map 5-methylcytosine (5 mc) patterns on a genome-wide scale. 

For sequence-specific information on DNA methylation, bisulphite-converted DNA is frequently used with a number of platforms. This process selectively converts unmethylated cytosines to uracil by the deamination of the cytosine (and subsequently thymidine if PCR-amplified). The methylated cytosines are resistant to this process [25]. Thus, specific changes are introduced in the DNA sequence that yield single-nucleotide resolution information about the methylation status of a segment of DNA. The resulting sequence differences between a methylated and unmethylated cytosine can be determined by direct sequencing [26]. Whole-genome bisulfite sequencing could be considered as the current gold standard for the genome wide identification of differentially methylated regions at a single nucleotide resolution. Unfortunately, the high quantitative and spatial resolution is cost-prohibitive, as it requires substantial sequencing to obtain a proper and even coverage. In addition to being unaffordable on a large scale, whole-genome bisulfite sequencing has its drawbacks of a low antibody resolution and low methyl binding protein enrichment of methylated regions [3].

Epigenotyping technologies such as the Infinium Human Methylation 27, 450, or 850 K BeadChip (Illumina Inc., San Diego, CA, USA) have emerged as alternative tools for DNA methylation-based biomarkers [3]. These technologies generate a methylation state-specific “pseudo-SNP” through bisulfite conversion, translating differences in the DNA methylation patterns into sequence differences that can be analyzed using quantitative genotyping methods [27]. These emerging technologies use highly standardized protocols that can be implemented with a degree of automation into existing genotyping pipelines and as such have the potential for large-scale, high-throughput studies [3]. As an example, the 450 K array provides a good compromise between coverage, throughput, cost, resolution, and accuracy, which are all vital factors in optimizing biomarkers and hence permitting epigenome-wide analysis to be positively adapted by the scientific community [28].

The other method of analysis includes enzymatic digestion with methylation-sensitive restriction enzymes and the capture of 5 mC by methylated DNA-binding proteins, followed by next-generation sequencing. Affinity purification, which is another method for DNA methylation detection, makes use of the methyl-binding domain, which binds to methylated CpG sites and measures the density of methylation in a given region. When combined with genomic DNA array or high-throughput sequencing, this technique can be used for genome-wide analyses. Unlike the bisulphite technique, however, this does not provide the base pair resolution [29].

The most novel technique, however, is deep sequencing. This provides a quantitative measure of methylation abundance rather than a relative measure, which is afforded by array-based techniques [30]. With the rapid advancement in better technologies and reduced costs, epigenetic-wide association studies (EWAS) are now increasingly performed in both experimental and clinical studies [31]. The availability of more advanced epigenomic platforms and data analysis tools, together with publicly available datasets, can be used to gain deeper insight into the pathologies of diabetes-associated kidney disease [32].

### 2.3. DNA Methylation in Diabetes-Associated Kidney Disease

DNA methylation may be a viable biomarker to predict disease progression in type 2 diabetes mellitus (T2DM). Toperoff et al. identified differentially methylated sites in T2DM and showed that low methylation levels at the analyzed site was an early marker of T2DM compared to controls, and that this effect was independent of the sequence polymorphism in the gene region [33]. Interestingly, a prospective study in an independent, young population cohort that demonstrated this same hypomethylation was able to identify the individuals that later progressed to T2DM [33]. 

There have been multiple studies that have evaluated the role of DNA methylation in diabetes-associated CKD [21,22,34,35]. Two different studies have used the Illumina Infinium HumanMethylation27 BeadChip (27 K Array) to assess the methylation status of over 27,000 CpG sites in patients with diabetes-associated CKD and diabetic end-stage kidney disease (ESKD) [21,22]. In one study of 96 controls and 96 participants with diabetes-associated CKD, 19 differentially methylated CpG sites were identified in genomic DNA extracted from whole blood [21]. Another group examined the differential methylation in DNA extracted from the saliva of 23 diabetic ESKD patients and 23 controls and discovered 187 genes containing more than one significantly differentially methylated CpG site [22]. Despite using the same technologies, no common genes were identified, attributable perhaps to the small samples sizes, different bio specimens on which the study was conducted, and different ethnic populations. 

In contrast, a well-powered epigenome-wide association study (EWAS) of eGFR and CKD investigated over 2000 participants from a diverse general population in each of the Atherosclerosis Risk in Communities (ARIC) Study and Framingham Heart Study to identify the epigenetic signatures of kidney function [12]. There were 19 CpG sites significantly associated with eGFR/CKD. Five of the 19 CpG sites were also associated with kidney fibrosis, as reflected in the biopsies of these individuals, as well as demonstrating DNA methylation changes in the kidney cortex [12]. This demonstrates that DNA methylation in blood is reflective of the methylation changes in other tissues. The authors identified *PTPN6* and the target genes of *CEBPB*, *EBF1*, and *EP300* as potential candidates for the gene regulatory mechanisms linking differential DNA methylation to kidney function in health and disease [12].

Others have identified far greater (1061) unique differentially methylated genes [23] in those with diabetes-associated CKD compared with the control. The use of renal tubule cells as opposed to genomic DNA from whole blood extracted in all previous EWAS may account for the larger number of significantly differentially methylated CpG sites in this study.

RUNX3 is a transcription factor that works in balance with the STAT5 transcription factor in the kidney fibrosis pathway [36]. *RUNX3* was the only unique gene that remained significantly differentially methylated, even after the application of a more stringent false discovery rate (FDR) correction, in two studies which analyzed the tubular epithelial cells [23] and peripheral blood [21] of participants with diabetes-associated CKD. This suggests that the altered DNA methylation and hence expression of *RUNX3* may lead to kidney fibrosis, which is a hallmark of progressive disease [37]. *PHB* is another gene that was again identified to be significantly differentially methylated by Ko et al. [23] and Swan et al. [38]. It has been involved in regulating transcription and apoptosis, and its dysregulation increases kidney fibrosis and oxidative stress, pathologic processes known to be involved in diabetes-associated CKD [37]. *MTHFR* is an example of another gene that has been proposed as an epigenetic biomarker in three studies, albeit with inconsistent results regarding its mechanism of influence [22,23,39]. Taken together, these data highlight the need for the consistency of potential DNA methylation markers in diabetes-associated kidney disease.

The functional implications of these significantly differential methylated genes have been investigated by several groups [23,34]. Functional gene analyses found that a proportion of the significantly methylated genes showed changes in gene expression [23]. Many of these genes, such as *TGFBR3* and *SMAD6*, belong to the TGF-β pathway, which is a well-known pathway for CKD development [23]. Unfortunately, however, no CpG sites or genes have been established as candidates to be taken forward in biomarker development [23,34].

In summary, whilst there is great interest in the role of DNA methylation as a potential biomarker in both the diagnosis and prognosis of diabetes-associated chronic kidney disease to date definitive epigenomic biomarkers are lacking. The spectrum of CKD across stages 1–5 adds to the complexity. Indeed, there is a need to identify specific methylation regions in diabetic CKD across all stages of the disease to serve as potential biomarkers of early versus late stages of diabetic CKD, but also to understand the role of genes in functional pathways. We have identified 239 CpG sites of differential DNA methylation between early and late diabetic CKD patients, as well as demonstrating the novel findings of a progressive loss of methylation (hypomethylation) of these CpG sites across all stages (1–5) of diabetic CKD. In the context of the identified 239 CpG sites, we have also identified associated genes (*CRISP2, PIWIL1*) which may have the potential to act as stage-specific diabetes-associated CKD markers (Lecamwasam A et al., forthcoming 2020) [40]. Understanding stage-dependent biomarkers and functional pathways may enable a deeper understanding of the mechanisms of the pathological process and consequently lead to a discovery of potential areas for therapeutic intervention to slow disease progression.

Research on the use of DNA methylation biomarkers, especially in the field of renal disease and diabetes-associated kidney disease, is still in its infancy. There are promising signs of advances in technologies, which cover increasing CpG sites and thus enable more precise assessments, increased interest in epigenetic epidemiology, and statistical approaches. There is a need for well-designed, longitudinal studies that involve phenotypes consisting of all stages of diabetes-associated CKD and ethnicities in adequately powered numbers, as well as the concomitant use of high-resolution DNA methylation technologies, in order to fully realize the potential of a DNA methylation as biomarker in complex diseases such as diabetes-associated CKD. 

## 3. Metabolomic Biomarkers in Diabetes-Associated Chronic Kidney Disease

The systematic analysis of small molecules termed metabolites, which are present in biologic specimens are considered to be final proxies for physiological homeostasis and gene-environment interactions and hence considered an increasingly useful tool for research in chronic kidney disease [41]. 

As with epigenetic biomarkers, the lack of reproducibility of results remains a weakness in metabolomic studies [42], and is often attributed to differences in patient demographics, different sample types, and variations in computational analysis [43,44]. In order to optimize the potential for metabolomics to be used as a biomarker, it is essential that studies report on when samples were taken and what pre-sampling checks were in place to limit variability [45]. In addition, the time between sample acquisition and sample processing must be stipulated, as this may increase the possibility of metabolite degradation and false-positive results [46]. Such is the sensitivity, every individual has their own unique metabolic phenotype that is subject to dynamic daily changes as a result of diet, medications, and diet–microbiome interactions [47]. Consequently, studies that rely on a single time-point sample have limited utility; rather, studies ideally need to be longitudinal and have at least two measurements over the study period in order to account for these dynamic changes [48]. 

One of the most salient confounders in metabolomic studies of chronic kidney disease is kidney function itself [49]. Kidney function, assessed as the glomerular filtration rate (GFR), is related to about one third of the detected metabolites in both general and CKD populations [50]. Illustrating the importance of the consideration of baseline GFR, Grams et al. showed a marked attenuation in the number of statistically significant metabolite associations when adjusted for baseline kidney function [49]. In addition, the classification of patients in CKD studies is usually based on their estimated GFR (eGFR) rather than the more accurate measured GFR. This limitation creates variability in the datasets [41]. However, it must be noted that even despite adjusting for kidney function, metabolites may vary due to changes in tubular secretion or resorption [49], in addition to being influenced by the diet, microbiome, and medications.

Even the best metabolomics platforms do not provide a complete coverage of the human metabolome, as they only detect less than one quarter of the known endogenous and exogenous metabolites in a given specimen [49]. Further complicating matters is the fact that different platforms, such as mass spectrometry (MS) compared to nuclear magnetic resonance imaging (NMR), provide a different coverage of the metabolome with varying sensitivities to different metabolites, making direct comparisons difficult [51]. Despite this, however, the stability and reproducibility of platforms such as liquid chromatography and gas chromatography coupled with mass spectrometry (LC-MS and GC-MS), as well as NMR spectroscopy, in large-scale cohort studies now exists for robust and high-quality data generation [52]. 

The Prevention of Renal and Vascular End-stage Disease (PREVEND) case-controlled study showed that patients with T2DM and norm albuminuria had no changes in their metabolite profile compared to those with microalbuminuria, who had lower levels of histidine and higher levels of the lipid butenoyl-carnitine in their plasma. Via mass spectrometry, the urine of patients with microalbuminuria was shown to have reduced levels of glutamine, tyrosine, and hexoses [53]. Another mass spectrometry study evaluating the urine metabolome in individuals with both Type 1 and Type 2 diabetes, with and without CKD, found differences in their metabolome [54]. Some of the metabolites which distinguished individuals with diabetes-associated CKD compared to diabetes alone include 3-hydroxyisovalerate, aconitate, citrate, 2-ethyl,3-hydroxypropionate, glycolate, 2-methylacetoacetate, and uracil [54]. A more recent NMR study showed that worsening glycaemic control affects the patterns of serum metabolites more strongly in early CKD [13]. As an example, increased levels of branched chain amino acids, tyrosine and formate, compared to lower levels of urea, creatinine, arginine, and pyruvate, were observed in individuals with early diabetes-associated CKD [13]. Other metabolites, such as arginine, methionine, and threonine, were identified as biomarkers of renal prognosis in a metabolomics study evaluating plasma samples obtained from the aortas and renal veins of patients with established CKD [55]. These data show that a single metabolomic biomarker in diabetes-associated CKD may be elusive, but perhaps a panel based on the ratios of identified altered metabolites may offer some potential [56]. 

Novel biomarkers will help guide therapy by distinguishing patients with progressive disease who may benefit from more aggressive medical therapy. Another potential beneficial effect is that biomarkers may help to differentiate treatment responders from non-responders. Compared to present day standards of a narrow set of biochemistry measures, metabolomics are instrumental in addressing such clinical questions, as these techniques enable a metabolic fingerprint that is unique to the individual and in turn may form the basis of personalized medicine. Some impressive studies supported by the National Institute of Health (NIH) illustrate how a patient’s metabolomic phenotype at baseline, prior to treatment, during treatment, and post treatment can inform about variations in responsiveness to medications and treatment outcomes in diabetes and cardiovascular disease [42]. This would be particularly useful knowledge, for instance, in individuals with diabetes-associated CKD who are refractory to treatment interventions and where the progressive, rapid decline in kidney function may necessitate earlier planning for renal replacement therapy [42]. Such metabolomic fingerprints will prove much more informative biomarkers than the current standards of kidney function, such as eGFR and urinary albumin [41].

In summary, a good study design is paramount in any research investigation, but particularly so in novel areas, such as the potential for metabolomic biomarkers, where there needs to be an emphasis on the consideration of potential confounders, the rigorous quality control of samples, and the use of ongoing advances in technology. In addition, collaborative work across multiple metabolomic platforms and metabolite profiling across large, longitudinal studies with multiple sampling time-points will have the potential to yield novel metabolomic biomarkers and therefore further insight into diabetes-associated pathophysiology and therapeutic opportunities in the future.

## 4. Gut Microbiome Biomarkers in Diabetes-Associated Chronic Kidney Disease

The normal gut microbiota influences the well-being of the host by contributing to its nutrition, metabolism, physiology, and immune function [57]. The disturbance of the normal gut microbiota (dysbiosis) has been implicated in the pathogenesis of obesity [58] and type 2 diabetes [59]. Preliminary evidence indicates that gut-derived metabolites, due to dysbiosis, may contribute to CKD and its related complications [60]. Individuals with CKD were shown to demonstrate lower numbers of Lactobacillaceae and Prevotellaceae families and 100-times higher *Enterobacteria* and *Enterococci* species [61]. The quantity of aerobic bacteria, including the *Enterococci* and *Enterobacteria* species, was higher in end-stage kidney disease (ESKD) than in healthy controls [62]. There is both a quantitative and qualitative gut microbiota imbalance in CKD, which is often accompanied with an increase in Lachnospiraceae, Enterobacteriaceae, and certain Ruminococcaceae and a reduction in some Prevotellaceae, Bacteroidaceae, *Lactobacillus*, and *Bifidobacterium* species [63]. In fact, Lun et al. identified that the genera *Lachnospira* and *Ruminococcus_gnavus* performed the best in differentiating between healthy controls and CKD [64]. In addition, they found that five phylotypes, including *Holdemanella*, *Megamonas*, *Prevotella2*, *Dielma*, and *Scardovia*, were indicators of the progression of CKD and haemodialysis [64]. In ESRD patients, there is a reduction in the absolute quantity of total bacteria with reductions in the beneficial butyrate-producing bacteria, such as *Roseburia* and *Faecalibacterium*, but enrichment with *Bacteroides* [65].

There is, however, a paucity of studies investigating the relationship of the microbiome across the specific stages of diabetes-associated CKD. A recent study by Wu et al. did demonstrate a change in the gut microbiota composition across different CKD disease stages in their discovery cohort of 30 controls and 92 participants with different stages of diabetic and hypertensive CKD [14]. They demonstrated a core CKD-associated microbiota consisting of seven genera and two species that were highly correlated with stages of CKD [14]. Importantly, they also showed that the gut-derived metabolites Indoxyl Sulphate (IS) and p-Cresyl Sulphate (pCS) were highest among advanced CKD patients, and the differences in the prediction of microbial gene functions in the metabolism of phenylalanine and tryptophan were significant between individuals without CKD and those with advanced CKD [14]. These data indicate the potential for specific gut microbes as biomarkers for early diabetes-associated CKD diagnosis and prognostication. 

Human microbiome investigations have currently reached a critical point where there is a move from bacterial description and investigation to an understanding of the mechanisms of action, opening the door for new clinical interventions [66]. These advancements have led to an increase in translational research in both the academic and private sectors [67]. As with the evolution of personalized medicine in metabolomics, a greater understanding of the gut microbiome has the potential to lead to new diagnostic biomarkers and therapeutics. Although we are yet to see these benefits in areas such as diabetes-associated CKD, bacterial probiotics are used clinically to enhance immune checkpoint blockade therapy for melanoma patients [68]. 

There are, however, limitations in clinical studies that need to be considered, such as the significant intra-individual variability in the microbiome due to host lifestyle and dietary changes; the reproducibility of results; underpowered case-control studies where the case and controls are sometimes phenotypically, aetiologically, and microbiologically heterogenous [69]. A common theme across the areas of epigenetics, metabolomics, and microbiome studies is the lack of statistically powered longitudinal and interventional studies involving study participants with well-defined diseases. This is especially important in the study design if we are to determine causality. Many of these microbiome studies are cross-sectional, single time point studies, and although they provide intriguing statistical associations [70], there is a need for longitudinal, prospective studies complemented by mechanistic experiments in animal models to establish whether a particular microbiome causes disease [67]. Furthermore, in most studies there is a paucity of detail on the effect of the microbiome and its products on potential molecular and functional pathways.

Compounding this is the problem that the majority of genes in human gut microbiomes cannot be functionally assigned, as the bacterial genome databases are incomplete [69]. Many clinical studies have used the targeted sequencing of 16S ribosomal RNA (rRNA) to survey the composition and structure of the gut, which is favored because it is economical; however, this is limited to assessment of bacterial taxonomic composition based on the sequence of only a single region of the bacterial genome [71]. Furthermore, the information on their metabolite effects is limited by the incomplete knowledge of bacterial genomic databases. However, PICRUSt (Phylogenetic Investigation of Communities by Reconstruction of Unobserved States) [72], an algorithm that uses 16S rRNA sequence data to predict the conserved bacterial functional capacity, permits in silico bacterial metagenomic analyses, thereby enabling more detailed functional annotations of microbial communities [59]. KEGG and MetaCyc are the most comprehensive databases for linking orthologous gene groups to reactions and metabolites [73]. 

It is important to understand the metabolic capabilities of the gut microbiota in order to appreciate their functions in human health and disease. As an example, microbial metabolites (branched chain amino acids) can serve as potential microbial biomarkers regarding metabolic disorders such as type 2 diabetes to prevent or mitigate the disease [74]. This adds further weight to the idea that the faecal microbiota richness is more a reflection of a temporal stage of the gut ecosystem development, and as an isolated biomarker does not provide information related to the gut microbiota stability and host health. Thus, focusing on the gut microbiota’s metabolic activity may be a better reflection of intervention success than the traditional microbiota richness or diversity [75]. 

Further studies in diabetes-associated chronic kidney disease should examine the diets and microbiome of individual participants and stratify patients based on their ethnicity, sex, and stage of CKD, as these are known potential confounders. Furthermore, investigations evaluating the functional implications of gut-derived metabolites are also important. Clinical trials are required to evaluate the effects of the manipulation of identified, specific microbes in the reduction in the levels of gut-derived metabolites or nephrotoxins and in improvements to the renal outcomes of these patients. In order to achieve a more effective combination of microbiome and metabolome for understanding the relevant metabolisms in chronic disease states such as diabetes-associated CKD, advanced multi-omic integration methods need to be developed. Finally, most studies have primarily focused on bacterial species rather than representing the functional interplay among the plethora of bacteria, archaea, viruses, fungi, and eukaryotes that all comprise the gut microbiome [69].

## 5. Concluding Remarks

Diabetes-associated kidney disease has significant global morbidity and mortality but, as yet, there is no definitive, prognostic novel biomarker or panels of biomarkers for routine use in clinical practice. Future directions should include more adequately powered population studies, assessed for changes in the epigenome, metabolome, and gut microbiome from a variety of readily accessible bio-specimens. These studies should ideally be longitudinal and cross-referenced with the diabetic CKD phenotype across all stages of kidney disease. This review offers insight into some potential novel biomarkers, Table 1, but is by no means an exhaustive compilation of all the novel biomarkers in diabetes-associated chronic kidney disease. As we transition into an era of personalized medicine, the potential integration of epigenetics, metabolomics, and the microbiome would indeed provide novel and invaluable insights into the pathophysiology and potential therapeutic opportunities for individuals with diabetes-associated CKD.

## Figures and Tables

**Table 1:** Summary of Potential Novel Biomarkers in Epigenetics, Metabolomics, and the Gut Microbiome Discussed in this Review.

Epigenetics	Metabolomics	Gut Microbiome
Differentially methylated genes with potential as biomarkers in diabetes-associated CKD.	Metabolites as potential biomarkers of diabetes-associated CKD prognosis.	Gut dysbiosis and gut-derived metabolites as potential biomarkers in diabetes-associated CKD.
*PTPN6*, *CEBPB*, *EBF1*, *EP300* [12]	3-hydroxyisovalerate, aconitate, citrate, 2-ethyl,3-hydroxypropionate, glycolate, 2-methylacetoacetate and uracil [53]	Reduced Lactobacillaceae and Prevotellaceae [60] Increased *Enterobacteria* and *Enterococci* [60]
*RUNX3* [35]	Tyrosine, formate [13]	Increased Indoxyl Sulphate (IS), p-Cresyl Sulphate (p-CS) [14]
*PHB* [22,37]	Arginine, methionine, threonine [54]	
*MTHFR* [21,22,38]		
*CRISP2* [39]		
